# The unique 3D arrangement of macrophage galactose lectin enables *Escherichia coli* lipopolysaccharide recognition through two distinct interfaces

**DOI:** 10.1093/pnasnexus/pgad310

**Published:** 2023-09-20

**Authors:** Massilia Abbas, Meriem Maalej, Ferran Nieto-Fabregat, Michel Thépaut, Jean-Philippe Kleman, Isabel Ayala, Antonio Molinaro, Jean-Pierre Simorre, Roberta Marchetti, Franck Fieschi, Cedric Laguri

**Affiliations:** Univ. Grenoble Alpes, CNRS, CEA, Institut de Biologie Structurale, Grenoble 38000, France; Univ. Grenoble Alpes, CNRS, CEA, Institut de Biologie Structurale, Grenoble 38000, France; Department of Chemical Sciences, University of Naples Federico II, Naples 80126, Italy; Department of Chemical Sciences, University of Naples Federico II, Naples 80126, Italy; Univ. Grenoble Alpes, CNRS, CEA, Institut de Biologie Structurale, Grenoble 38000, France; Univ. Grenoble Alpes, CNRS, CEA, Institut de Biologie Structurale, Grenoble 38000, France; Univ. Grenoble Alpes, CNRS, CEA, Institut de Biologie Structurale, Grenoble 38000, France; Department of Chemical Sciences, University of Naples Federico II, Naples 80126, Italy; Univ. Grenoble Alpes, CNRS, CEA, Institut de Biologie Structurale, Grenoble 38000, France; Department of Chemical Sciences, University of Naples Federico II, Naples 80126, Italy; Univ. Grenoble Alpes, CNRS, CEA, Institut de Biologie Structurale, Grenoble 38000, France; Institut Universitaire de France (IUF), Paris, France; Univ. Grenoble Alpes, CNRS, CEA, Institut de Biologie Structurale, Grenoble 38000, France

## Abstract

Lipopolysaccharides are a hallmark of gram-negative bacteria, and their presence at the cell surface is key for bacterial integrity. As surface-exposed components, they are recognized by immunity C-type lectin receptors present on antigen-presenting cells. Human macrophage galactose lectin binds *Escherichia coli* surface that presents a specific glycan motif. Nevertheless, this high-affinity interaction occurs regardless of the integrity of its canonical calcium-dependent glycan-binding site. NMR of macrophage galactose-type lectin (MGL) carbohydrate recognition domain and complete extracellular domain revealed a glycan-binding site opposite to the canonical site. A model of trimeric macrophage galactose lectin was determined based on a combination of small-angle X-ray scattering and AlphaFold. A disulfide bond positions the carbohydrate recognition domain perpendicular to the coiled-coil domain. This unique configuration for a C-type lectin orients the six glycan sites of MGL in an ideal position to bind lipopolysaccharides at the bacterial surface with high avidity.

**Significance Statement**

The surface of bacteria is a marker of their presence when invading a host, and gram-negative types are decorated with lipopolysaccharide (LPS) carbohydrates. In this report, the recognition of LPS from *Escherichia coli* bacteria by a sugar-binding protein (lectin) present at the surface of human immune cells is described. Using a multidisciplinary approach, the presence of an unforeseen sugar-binding site at the surface of the protein was demonstrated. The tridimensional arrangement of the lectin, determined by a combination of bioinformatics and structural biology methods, explains how its sugar-binding sites allow a very strong binding to the bacterial surface. These findings illustrate how this immunity protein can recognize pathogenic bacteria with very diverse carbohydrates at their surface.

## Introduction

**Fig. 1.**
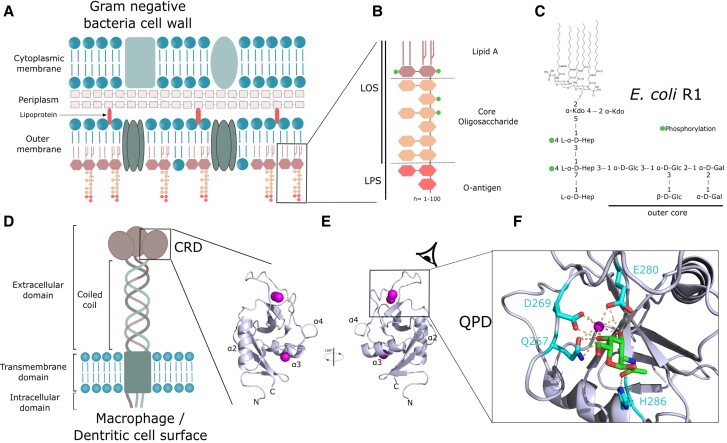
Organization of gram-negative bacteria cell wall and of MGL. A) General structure of gram-negative bacteria cell wall. B) LPS composing the outer leaflet of the outer membrane. C) Structure of *E. coli* R1 LOS mainly used in this study. D) Domain organization of MGL at the surface of antigen-presenting cells. E) Structure of CRD domain of MGL. F) Close-up view on GalNAc sugar bound to the calcium-binding site (PDB:6PY1). Calcium ions are shown as spheres.

The outer membrane of gram-negative bacteria is compositionally asymmetric with lipopolysaccharides (LPSs) covering most of its surface (Fig. 1A), while phospholipids compose the inner leaflet. LPSs form a highly impermeable barrier and are critical in bacterial virulence (1); their structural variability and tight assembly protect bacteria against uptake of antimicrobials and enable evasion from host defenses. Constant transport and maintenance of LPS in the outer membrane are critical in the survival of bacteria. LPSs are composed of three moieties: the lipid A formed by *N*- and *O*-acylated di-glucosamine, the core oligosaccharide (core OS), and O-antigen polysaccharide repeat (Fig. 1B). These complex glycolipids are detected by the immune system through the lipid A via the well-described LBP-MD2-TLR4 cascade (2) and by the caspase system in the cytoplasm (3). Antibodies directed against the glycan moieties, core OS (4), and O-antigen polysaccharides are also produced by the immune system to modulate bacterial infections (5). Another protein family present on antigen-presenting cells, C-type lectin receptors (CLRs), has been shown to bind sugars from the core OS of LPS (6–8). CLRs are key immunity receptors, which recognize a plethora of pathogen glycans (9), and the interaction of these CLRs with their ligands, discriminating nonself from self-molecular motifs, allows dendritic cells to modulate the immune response toward either activation or tolerance (10). Macrophage galactose-type lectin (MGL) is a trimeric type II CLR expressed on the cell surface of macrophages and dendritic cells (Fig. 1D). It mediates interactions between endothelial and cancer cells (11) but also recognizes microbial glycans. Its main role appears to be an immunomodulatory activity, reducing excessive inflammatory responses. So far, MGL has been described to recognize *Staphylococcus aureus*, *Campylobacter jejuni*, *Klebsiella pneumoniae*, *Neisseria gonorrhoeae*, *Bordetella pertussis*, and *Mycobacterium tuberculosis* (12–15).

MGL is a transmembrane protein composed of an intracellular signaling domain, a transmembrane domain, a coiled-coil trimerization domain, and a C-terminal carbohydrate recognition domain (CRD) (Fig. 1D). The CRD fold is highly conserved in C-type lectins and is organized as a double-loop structure (Fig. 1E) stabilized by at least two conserved disulfide bridges. The overall domain is a huge loop in itself with its N and C terminus joined together, thanks to the first disulfide bridge, which contains another loop (the so-called long loop region) also stabilized by the second conserved cysteine bridge (16). Some C-type lectin domains, including MGL, possess an additional N-terminal β-hairpin that is stabilized by a third cysteine bridge conserved in these long-form subtypes of CRDs. The domain presents a mixed α/β-fold and a large proportion of loops with undefined secondary structures (Fig. 1E). For most of the CLRs reported, glycan-binding site is calcium dependent and characterized by a tripeptide motif (EPN/QPD) and residues from the adjacent β-strand that assume metal coordination (17). MGL possesses a QPD (267–269) motif characteristic of recognition of glycans with terminal galactoses (Fig. 1F). The X-ray structure of human MGL-CRD (18) in complex with galactose-containing ligands shows two galactose ring hydroxyl groups 3 and 4 bound to the calcium ion. Additionally, H286 is proposed to be responsible for selectivity toward *N*-acetyl through a water-mediated hydrogen bond (19). MGL binds preferentially to terminal *N*-acetylgalactosamine residue and presents, for a C-type lectin, an unusually low (µM) dissociation constant for the monosaccharide (20). The interaction of MGL with terminal galactoses from the core OS was shown for *C. jejuni* LPS (14) and for *E. coli* R1 type core OS (Fig. 1C) (21).

In this work, we have investigated MGL binding to OSs isolated from deacylated LPS or to native LPS directly exposed on whole cells. Our results show that in the trimeric oligomerized form, the CRD of MGL adopts a specific 3D arrangement that allows a unique presentation of its six glycan-binding sites (two per CRD), composed of the canonical QPD calcium-binding motif and a newly described interaction site.

## Results

### MGL extracellular domain strongly binds to bacterial surface, independently of the QPD motif

**Fig. 2.**
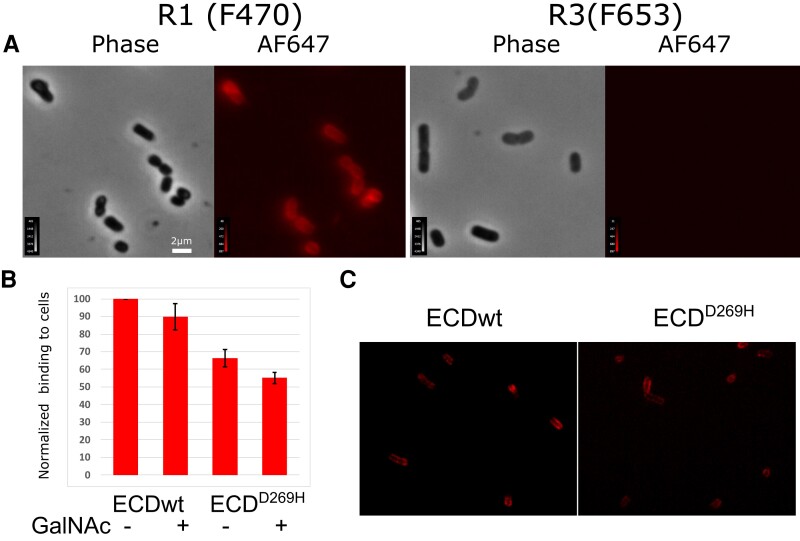
MGL-ECD binds specifically to R1 presenting *E. coli* cells independently of the QPD motif. A) Phase contrast and epifluorescence microscopy images of AF647-labeled ECD incubated with R1 (left) or R3 (right) presenting *E. coli* cells. B) Flow cytometry quantification of MGL-ECD wt and D269H variant labeled with AF647 bound to R1 cells in the presence or absence of 10 mM GalNAc competitor. C) Confocal fluorescence image corresponding to conditions in B), showing strong MGL-ECD^D269H^ binding to cells.

MGL extracellular domain (ECD) was shown by NMR to interact with the terminal galactoses of *E. coli* R1 type core OS. To establish MGL binding in the context of R1 OS assembled at the cell surface, interaction of MGL-ECD was tested with live bacteria. *Escherichia coli* bacteria exhibit variable structures of the core OS, so we chose to compare R1 and R3 types (Fig. 1C; Fig. S1) because they represent together more than 80% of *E. coli* strains including enterohemorrhagic species (22). Two bacterial strains carrying R1 and R3 core OS structures but no O-antigen, respectively F470 and F653, were thus compared for MGL interaction. MGL-ECD was labeled with Alexa Fluor 647 (AF647), incubated with *E. coli* bacteria, and excess protein was washed. Bacteria were imaged by fluorescence microscopy. F470 bacteria were significantly labeled at their surface by MGL while F653 showed no labeling, confirming that MGL can recognize R1 core OS on cells (Fig. 2A). In order to ascertain that the interaction with the LPS observed was specific, the interaction with R1 cells was reproduced in presence of 10 mM GalNAc, that possess a low micromolar affinity for MGL, as a competitor and quantitatively assessed MGL binding by flow cytometry (Fig. 2B; Fig. S2). We found that GalNAc at high concentration could not significantly compete to the binding of MGL to R1 presenting cells.

The inability of the GalNAc monosaccharide to compete with the MGL binding to F470 cells could be ascribed to the multivalency of the interaction between the MGL trimer and R1 OSs presented on the cell surface. We thus designed a mutant of a key residue of MGL-CRD that would abolish MGL carbohydrate-binding capacity. D269, part of the conserved QPD motif (Fig. 1F), is involved in calcium-mediated binding of GalNAc to MGL (20), so we decided to produce a D269H mutant to have a steric and electrostatic inhibition of the interaction with Ca^2+^ ion in canonical carbohydrate-binding site. MGL-ECD^D269H^ labeled with AF647 was thus incubated with F470 cells and imaged (Fig. 2C). We surprisingly found that MGL^D269H^ was still able to significantly bind bacteria. This was quantified by flow cytometry that showed only a 30% decrease in binding of the D269H variant to cells (Fig. 2B; Fig. S2) with little additive effect upon addition of 10 mM GalNAc. The behavior of this variant and the inability of GalNAc to inhibit significantly the binding suggest that, while the QPD motif is contributing to the interaction with R1 at the cell surface, it is not the main determinant of the interaction.

MGL strongly binds to R1 core OS on cells and, while the integrity of the QPD motif contributes to the interaction with R1 core OS, it is not the main determinant of the interaction. We thus hypothesized the existence of a secondary glycan-binding site in MGL and investigated its localization by NMR.

### MGL-CRD binds to LPS-derived OSs through a new binding surface

MGL-CRD and its binding to GalNAc and tumor-associated glycopeptides were previously characterized by NMR, X-ray crystallography, and molecular dynamics. Those studies show a clear involvement of the QPD motif, with a particular contribution of H286 in the recognition of the *N*-acetyl moiety (18, 20). MGL-CRD^wt^ and MGL-CRD^D269H^ have been produced and analyzed by ^1^H-^15^N NMR spectroscopy to localize the binding site of LPS-derived OS. Wild-type MGL-CRD shows a spectrum similar to the one already published. D269H variant ^1^H-^15^N correlation spectrum is also characteristic of a well-folded protein and comparable to the wild-type spectrum (Fig. S3). Backbone resonances of wild-type and D269H variant were assigned and used to predict their secondary structure content. It confirmed that MGL-CRD^D269H^ contains the same secondary structure elements than the wild-type protein (Fig. S3). The mutation, by abolishing the proper coordination of the calcium ion, probably destabilizes the whole GalNAc-binding site. Therefore, the resonances from residues 265–282 remained unassigned in D269H variant.

**Fig. 3.**
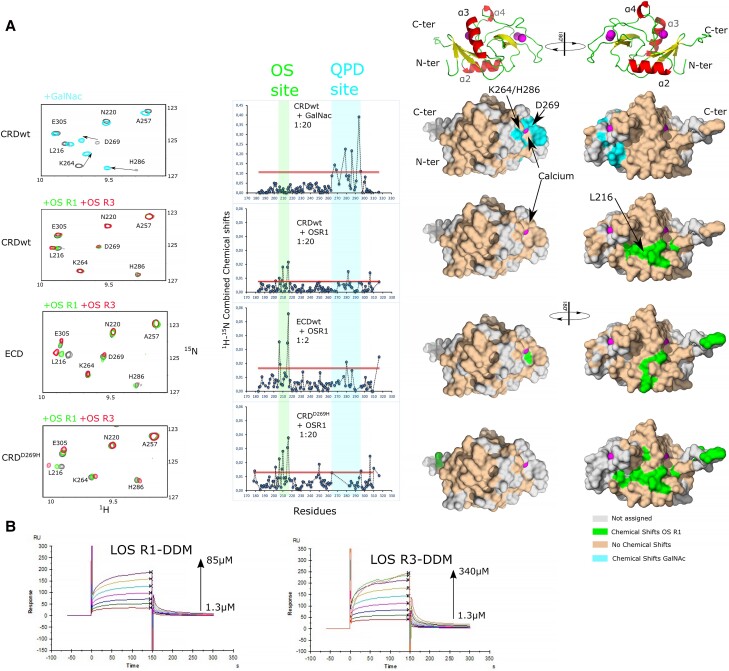
GalNAc- and LPS-derived OSs interact on two opposite surfaces of MGL. A) Left: extracts of ^1^H-^15^N correlation spectra of the CRD, CRD^D269H^, and the CRD in the full ECD upon interaction with GalNAc, OS R1, or OS R3. Middle: CSP of the corresponding interactions represented with respect to amino acid sequence. The red line marks the threshold of significant CSP. Right: Significant CSP represented on the CRD surface upon interaction with GalNAc or OS R1. B) SPR interaction of LOS R1 and LOS R3 in detergent micelles with immobilized MGL-ECD.

First, the binding to GalNAc sugar was assessed for both proteins. 2D ^1^H-^15^N correlation experiments show resonances, each one corresponding to the amide frequencies of individual amino acids. Addition of a ligand perturbs the amide frequencies at the vicinity of the binding site and can be good reporters of both the affinity and the amino acids involved in the binding. As reported by Diniz et al. (20) MGL-CRD binds strongly to GalNAc in the characterized binding site between residues 264 and 296, with strong chemical shift perturbations (CSPs) of D269 and H286 amide resonances (Fig. 3A; Fig. S4). MGL-CRD^D269H^, as predicted, does not show any CSP upon binding to GalNAc (Fig. S5), consistent with its inability to bind to GalNAc affinity column during purification.

Lipooligosaccharides (LOS) assemble into large vesicles in solution that rapidly sediment and are not suitable to perform interactions by NMR. Soluble LOS-derived OSs of R1 and R3 types (Fig. 3B; Fig. S1) were then produced by chemical deacylation of LOS (21). The interaction of CRD^wt^ and CRD^D269H^ was then tested with R1 and R3 OSs (Fig. 3; Figs. S4, S5, and S8). Interaction with OS R1 showed CSP of the CRD^wt^^1^H-^15^N resonances on a fast exchange regime with respect to NMR timescale with no saturation of the binding even at high OS concentration, suggesting a weak affinity (*K*_d_ ≥ 5 mM). Furthermore, residues of the CRD experiencing high CSP upon OS R1 binding lie on a surface opposite to the GalNAc-binding site, in green in Fig. 3A, and involve residues 202–216 around the α2 helix. The same interaction performed with the D269H variant showed a very similar interaction site opposite from the QPD motif. We thus postulate that the new interface perturbed by OS R1 is responsible for the binding of MGL to F470 *E. coli*. As a control, we also tested the binding of the CRD^wt^ and D269H variant to OS R3 in the same conditions (Fig. 3; Fig. S8). OS R3 caused very similar CSP at the surface of CRD^wt^ and CRD^D269H^. The new interaction surface of MGL involved in glycan binding does not show specificity for R1 core OS on the contrary to results obtained on cells. The configuration of the NMR interactions is very different from the in vivo experiments; the CRD domain is used instead of the ECD, and the OSs are free in solution and are not presented on the cell surface as multivalent ligands. In order to confirm that the binding observed on the isolated CRD also applies to the CRD in the context of the trimeric ECD, we investigated the ECD by NMR.

The ECD of MGL is a large protein (homotrimer of 84 kDa) for NMR spectroscopy due to signal broadening arising for long molecular tumbling correlation times. The protein was thus expressed and purified as a perdeuterated version. ^1^H-^15^N correlation spectrum of ^2^H,^15^N-labeled MGL-ECD is of high quality considering the protein size and elongated shape and is characteristic of a well-folded protein. When comparing the ^1^H-^15^N resonances observed on spectra recorded with isolated CRD, it is apparent that the footprint of the CRD domain is present in the ECD of MGL (Fig. S6). Several additional overlapped resonances can be observed around 8.2 ppm in the proton dimension and probably arise from the coiled-coil domain. The low stability (several days) of MGL-ECD at the temperature needed to record NMR spectra (above 35°C) did not allow its de novo assignment by backbone assignment experiments. The good ^1^H and ^15^N agreement between CRD signals in ECD and isolated CRD permitted the transfer of most assignments from CRD to ECD (Fig. S6). MGL-ECD was thus titrated with increasing concentrations of OS R1 and OS R3. CSPs induced by the interaction with R1 or R3 OS are very similar to those observed with isolated CRD, though the surface involved is not as extended (Fig. 3A; Fig. S7). This suggests that the assembly of the CRD domain in the full-length ECD has no influence on the selectivity of MGL toward either R1 or R3 chemical structure when interaction occurs with isolated OSs. To confirm these observations with an alternative method, R1 and R3 LOS were solubilized in dodecylmaltoside (DDM) detergent micelles and flowed over MGL-ECD specifically oriented by immobilization through its N terminus by surface plasmon resonance (SPR) (Fig. 3B; Fig. S7). Fitting of the respective sensorgrams at equilibrium produced an apparent affinity constant of about 15 µM for both R1 and R3 LOS, confirming the lack of specificity toward the OSs when not presented as a surface.

The presence of two different glycan-binding sites at the surface of the MGL-CRD on two opposite surfaces is unprecedented in C-type lectins. It suggests that in the ECD, both sites are accessible to bind their ligands, and we thus investigated the global arrangement of the CRDs in MGL-ECD.

### MGL-CRDs are oriented perpendicular to the coiled-coil domain and can present six sugar-binding sites to bacterial surfaces

**Fig. 4.**
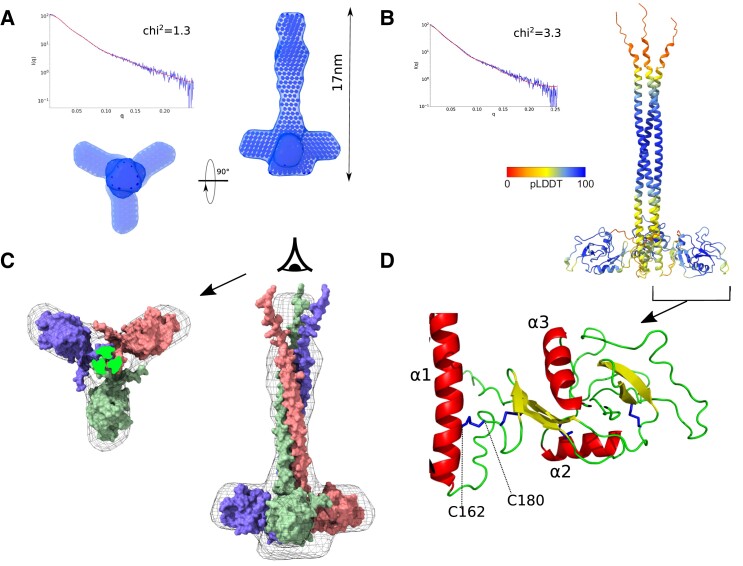
Combined SAX–AlphaFold model of MGL-ECD. A) SAXS of MGL-ECD with SAXS curve (top left blue) and the corresponding fit (red) of the SAXS envelope, calculated from *P*(*r*) distribution, shown as surface from side and N terminus of coiled-coil view. B) AlphaFold model with the best correspondence to SAXS curve, colored by pLDDT score. The calculated SAXS curve of this model is shown (red) compared with the experimental curve (blue). C) Best AlphaFold model of MGL-ECD adjusted into the SAXS envelope (in mesh) in side and from the N terminus of coiled-coil view. D) Close-up view on the C162–C180 disulfide bond orienting the CRD in the best AlphaFold model.

The structure of MGL-ECD is unknown, and we studied its overall structure by small-angle X-ray scattering (SAXS). This method enables to assess the size and shape of a macromolecule in solution, at a low resolution. MGL-ECD SAXS curve confirms the presence of a trimeric protein with an estimated molecular weight (MW) of 94 kDa (vs 84 kDa theoretical MW) and a gyration radius of 5.6 nm, suggesting an elongated protein (23). Calculation of pairwise distribution, *P*(*r*), showed a maximum interatomic distance of 17 nm (Fig. S9) consistent with the expected elongated shape of the ECD. *P*(*r*) was used to calculate an envelope of MGL-ECD (see Materials and methods section). The envelope (Fig. 4A) is characterized by an elongated structure, corresponding to the coiled-coil domain, with three large bulges on its side that can be ascribed to the CRDs. The SAXS-derived envelope does not allow to orient at an atomic scale the CRD, but the location of the bulges suggests that the CRD domains are perpendicular to the coiled-coil domain. This orientation would be significantly different from an about 120° angle observed between coiled-coil and CRD domain observed for langerin or MBP trimers (24, 25).

To position the CRD into the SAXS envelope, MGL-ECD models were generated with the AlphaFold structure prediction protocol (26, 27) (see Materials and methods section). This method has provided atomic-scale prediction of protein structure of unprecedented accuracy with a combination of machine learning and evolutionary data. The models show a long N-terminal coiled-coil domain (N86-N169) followed by the CRD (C181-H316). The arrangement of the CRD relative to the coiled-coil domain is variable and allows to sort the models into two clusters. The lack of well-defined interdomain contacts can be explained by low AlphaFold per residue score (pLDDT) at the interface and little interactions predicted in the prediction alignment error matrix (Fig. S10). One new disulfide bond is nevertheless predicted in all models between coiled-coil (C162) and CRD (C180) (Fig. 4D; Fig. S10). This disulfide bond is consistent with mass spectrometry analysis of MGL-ECD, which displays an 8 Da difference with the theoretical mass, corresponding to a total of four disulfide bonds (Fig. S11). The two cysteines involved are also strictly conserved in the MGL family in mammals (Fig. S12; Table S1), and the disulfide bond at the corresponding position was shown experimentally in the homologous protein asialoglycoprotein receptor 1 (28).

The two clusters of models are different in the orientation of the CRD domains with an almost 180° rotation around the C160–C182 disulfide bond (Fig. S10). To determine which cluster corresponds better to the conformation in solution, models were evaluated against experimental SAXS data. SAXS curves were back-calculated from the models and compared with the experimental one (Fig. 4B; Table S2 and Fig. S13). Cluster 2 structures show systematically a better fit compared with cluster 1. The best matching structures of each cluster can also be inspected visually by adjusting the structures into the SAXS-derived envelope (Fig. 4C; Fig. S14). The cluster 2 models are in the best accord to the SAXS data, and the structure with the lowest *χ*^2^ with respect to the SAXS curve was retained for analysis (Fig. 4B–D).

**Fig. 5.**
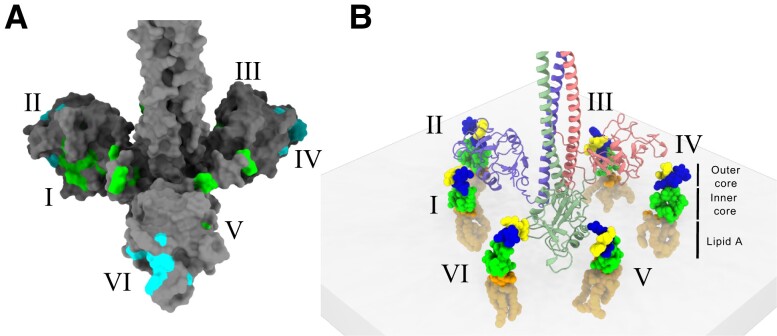
Combination of SAXS and AlphaFold defines a CRD arrangement that presents up to six accessible glycan-binding sites (I to VI). A) Representation of the two glycan-binding sites, with the NMR CSP of GalNAc (cyan) and OS R1 (green) on the best SAXS–AlphaFold MGL structure. B) Schematic view of six R1 LOS molecules, with an orientation similar to what is found at the bacterial surface, facing the six glycan-binding sites of MGL-ECD.

The two glycan-binding sites of the CRD, the canonical QPD motif and the newly described OS-binding site, can be represented on the surface of the MGL model (Fig. 5A) in cyan and green, respectively. The orientation of the CRD is such that QPD and OS sites from two neighboring CRDs face each other. If we consider that the most likely configuration of MGL binding to the bacterial surface would be perpendicular to the membrane, the CRDs are able to present up to six glycan-binding sites to LPS core OS (Fig. 5B). In that configuration, even if the affinity of MGL for isolated core OS is low, the avidity of the interaction would ensure a tight binding to the surface, consistent with our observations on bacteria presenting R1 core OS.

## Discussion

Glycoconjugates are present at the surface of most cells, as well as in extracellular matrices and biofilms. In complex multicellular organisms, the sugar environment is very rich and heterogeneous. The immune system must recognize friends from foes and clear pathogens but also tailor its response to avoid excessive inflammatory response. The recognition of pathogens vs commensals is critical and also relies on subtle variations of microbial glycome. MGL has been reported so far to recognize several bacterial pathogens, with different cell wall structures, through their surface glycans.

**Fig. 6.**
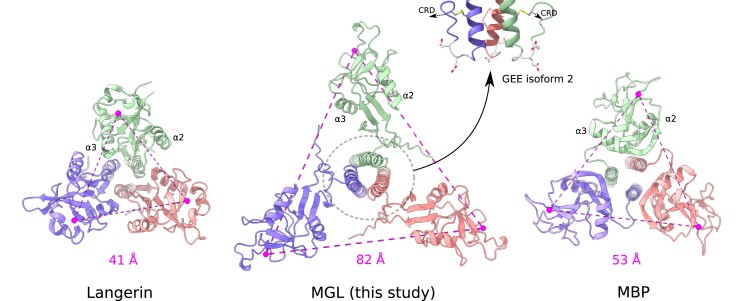
Comparison of the MGL model with other trimeric C-type lectins. The MGL model, langerin (PDB:3KQG), and mannose-binding protein A (PDB:1KWW) are shown from the C-terminal side of the coiled-coil domain. The calcium ions of the canonical-binding sites are indicated as well as the distances in magenta between adjacent sites. The C terminus of the coiled-coil domain of MGL is surface accessible (gray circle), and the MGL isoform 2 studied here has an additional GEE tripeptide (gray). The C162–C180 disulfide bond is in yellow.

While MGL attaches strongly to *E. coli* surface presenting R1 type core OS, this binding is largely independent of the QPD GalNAc-binding site. We could show that a second interface, opposite to the QPD-binding site, binds LPS core OS. Several examples exist of secondary binding sites in C-type lectins. They can be located adjacent to the conserved calcium-binding site to extend the binding interface and confer specificity toward a given ligand like observed for trehalose dimycolates for Mincle (29) or on a more remote site like for heparin for langerin (30) and through cooperativity for DC-SIGN (31, 32). The presence of a second binding site completely opposite to the canonical binding site is nevertheless unusual. We suggest that this is correlated with the peculiar 3D arrangement of MGL-CRDs compared with other multimeric C-type lectins. Other trimeric C-type lectins like langerin or mannose-binding protein (MBP) (24, 25) adopt a compact arrangement of their CRDs (Fig. 6) with their canonical binding sites accessible at the extremity of the proteins. Their calcium-binding sites lie within 50 Å of each other compared with about 80 Å for MGL. It allows MGL to target surfaces with much distant glycan epitope. Furthermore, this extended conformation makes the C-terminal loop of the coiled-coil neck domain, connecting it to the CRD, accessible at the surface and could contribute to glycan binding (Fig. 6). This region of the protein varies between isoforms 1 and 2 of human MGL (Fig. 6; Fig. S12) with insertion of three additional residues (G171–E172–E173) in isoform 2 (this study). These residues could participate to the interaction of MGL with a bacterial surface but could also be important for the orientation of the CRD. The reduction of the coiled-coil CRD linker in isoform 1 would alter, in turn, the orientation of the CRDs by likely leading them to rise upward. Thus, while these three residues’ insertion, from isoform 1 to 2 of MGL, does not modify the glycan-binding specificities of their CRDs, it might impact drastically the relative geometry of the CRDs in both trimeric isoform and thus their specificity toward different glycan landscape. The conserved disulfide bond positions the CRD domain perpendicular to the coiled-coil axis and has important implications with respect to glycan binding. As we have recently shown on another CLR, thanks to molecular dynamic studies, DC-SIGN can adapt to various distance distribution of glycan epitope presentation, thanks to a rather large flexibility between the neck and the CRD domains (33). Here, a different situation occurs in the case of MGL. The presence of the newly identified C162–C180 bridge strongly constrains the extension capabilities of CRDs from the neck (Fig. 4D). However, the CRD domains show here no extensive contacts with the coiled-coiled neck domain, and subtle variations of the CRD orientation through rotation around the disulfide bond axis might allow plasticity in the presentation of the binding sites. However, the limitation in distance is compensated here, in MGL, by the presence of the additional noncanonical OS-binding site on the opposite side, within the CRD, of the Ca^2+^-dependent QPD site. This, combined to CRD subtle rotation, might provide a large set of potential adaptation to different surfaces.

Here, the CRD orientation makes both QPD- and OS-binding sites accessible for binding glycans assembled on a surface. The presence of these six binding sites highly increases the multivalency of the interaction and probably explains the very broad pathogen-associated molecular patterns that MGL is capable to recognize, from both gram-positive and gram-negative bacteria, as well as *M. tuberculosis* (12–15). We can hypothesize that the mode of recognition by MGL of teichoic acids, which are polysaccharides assembled similarly as LPS at the surface of *S. aureus* (12), resembles that of LPS.

The 3D arrangement of MGL can explain its recognition of different cell surface glycans. Nevertheless, its binding to R1 and R3 core OS highly differs between isolated ligands and ligands presented at the cell surface. In our experimental conditions, cells are extensively washed with buffer before imaging. Thus, only the very stable interaction with R1 core OS is detected, and we hypothesize that the interaction of MGL with R3-producing cells is more labile. The avidity between MGL and *E. coli* surfaces presenting R1 is certainly key to the interaction. As already shown for MGL, a 150-fold affinity increase is measured between CRD and ECD binding to GalNAc-conjugated bovine serum albumin (BSA) (11). While the difference in avidity toward R1 or R3 might be linked to the protein, it should also be considered that the presentation of the LOS core OS on the surface of cells might differ. Furthermore, it is still unknown how the strength of the interaction of MGL with R1 or R3 presenting bacteria will relate to the function of the immune cell recognition and how it will, in turn, affect the adhesion, signaling, or antigen uptake.

So far, we have examined the binding of MGL to LPS that do not contain O-antigens. Most clinically relevant gram-negative bacteria possess O-antigen of very variable compositions and length (34). This dense and long (∼10–40 nm) layer of polysaccharides could be either recognized by MGL, thanks to its ability to bind various glycans, or, on the other hand, it could block access to the core OS and prevent recognition. This should be the focus of future studies on the role of MGL in the recognition of gram-negative bacteria and the subsequent implications in the regulation of the immune response.

## Materials and methods

### Protein expression and purification

Human MGL isoform 2 ECD (residues Q85-H316 Uniprot Q8IUN9-2) with an N-terminal Strep-tag II and a factor Xa cleavage site (MASWSHPQFEKIEGRGGG) was expressed and purified as already reported (21). Briefly, MGL-ECD was over-expressed in *E. coli* BL21(DE3) cells in inclusion bodies. Inclusion bodies were solubilized in guanidine buffer (25 mM Tris, pH 8, 150 mM NaCl, 6 M guanidine, and 0.01% B-mercaptoethanol). MGL-ECD was subsequently refolded using a drop-by-drop dilution in renaturation buffer (100 mM Tris, pH 8, 150 mM NaCl, and 25 mM CaCl_2_) and was subjected to two purification steps: a GalNAc–agarose affinity column (Sigma), eluted with EDTA buffer (150 mM NaCl, 25 mm Tris, pH 8, and 10 mM EDTA) followed by a Toyopearl HW-50S gel filtration column (Tosoh Bioscience). MGL-ECD was also produced as perdeuterated ^2^H,^15^N-labeled form ([U-^2^H,^15^N] MGL-ECD) in 95% D_2_O with d-glucose-d7 as glucose source as described (35). MGL-ECD^D269H^ mutant was over-expressed in *E. coli* BL21(DE3) cells in LB medium as inclusion bodies, which were subjected to the same solubilization, and renaturation steps described above. MGL-ECD^D269H^ was purified using an AktaXpress with a Strep-tag affinity column eluted with 2.5 mM desthiobiotin followed by a Toyopearl HW-50S gel filtration column (Tosoh Bioscience).

MGL-CRD and MGL-CRD^D269H^ (C181–H316 Uniprot Q8IUN9-2) with N-terminal His-tag and TEV cleavage site (HHHHHHIEGRGGGGG) were expressed and purified as described (11) in M9 minimal medium as ^13^C,^15^N-labeled proteins. An MGL-CRD^D269H^ binding assay was performed on the GalNAc–agarose affinity column used for ECD purification to assess its affinity for GalNAc, which revealed it did not bind to GalNAc affinity column.

### Fluorescence microscopy and flow cytometry

MGL-ECD and MGL-ECD^D269H^ were labeled with Alexa Fluor 647-NHS (Invitrogen). Briefly, MGL at 5 mg/mL in PBS buffer was incubated in 200 mM sodium bicarbonate and 0.4 mg/mL AF647-NHS for 1 h. Excess dye was removed with G25-PD10 desalting column (GE Healthcare), and MGL fractions dialyzed further against PBS buffer and concentrated. *Escherichia coli* R1 bacteria carrying R1 core OS (F470, derivative from *E. coli* O8:K27) and R3 (F653, derivative from *E. coli* O14:K7) (22) were grown in LB at 37°C under agitation up to 0.9 OD_600_ _nm_. Cells were collected by centrifugation, washed in cold PBS, and incubated with 670 nM MGL-AF647 in PBS and 2 mM CaCl_2_ buffer for 15 min. Cells were washed five times with cold PBS and imaged. For each sample, 2 μL of cells in suspension was mounted between a glass slide and a 1.5H 170 µm thick glass coverslip and observed using an inverted IX83 microscope, with the UPLFLN 100× oil immersion objective from Olympus (numerical aperture 1.49), using a fibered Xcite Metal-Halide excitation lamp in conjunction with the appropriate excitation filters, dichroic mirrors, and emission filters specific for AF647 (4X4MB set, Semrock). Acquisitions were performed with Volocity software (Quorum Technologies) using a sCMOS 2,048 × 2,048 camera (Hamamatsu ORCA Flash 4, 16 bits/pixel) achieving a final magnification of 64 nm per pixel.

Flow cytometry was performed on a VYB device (Miltenyi biotech) and analyzed with Macsquant software. Cells (50 µL) grown in LB at DO_600_ _nm_ = 1 were resuspended in presence of 670 nM MGL-AF647 (wt or D269H variant) in PBS, 2 mM CaCl_2_ with/without 10 mM GalNAc for 15 min, centrifuged twice to remove excess protein, resuspended in 150 µL, and injected for FACS analysis until 200,000 events were recorded. MGL-ECD binding to cells was expressed as % population × mean fluorescence (cy5 channel) and normalized to 100% for MGL-ECD wt binding.

### LOS and OS preparation

F470 and F653 cells were grown in LB. LOS were extracted following the phenol–chloroform–petroleum ether (PCP) method and de-*N*- and *O*-acylated as already described (21, 36). LOS R1 (0.84 mM) and LOS R3 (0.6 mM) were solubilized in DDM micelles by addition of 150 mM of DDM in HBS-N and 2 mM CaCl_2_ for 15 min. Insoluble material was discarded by ultracentrifugation at 100,000 *g* for 30 min, and sample homogeneity was checked by dynamic light scattering.

### SPR experiments

SPR interaction was performed using oriented surfaces of ECD-MGL, specifically N-terminally biotinylated, thanks to a sortagging procedure (37). Streptavidin at 100 µg/mL in 10 mM NaOAc, pH 4, was immobilized on sensor chip S Serie CM3 (Cytiva). Biot-ECD was diluted at 0.5 µg/mL in running buffer (HBS-N [cytiva], 2 mM CaCl_2_, and 300 µM DDM) and injected at 5 µL/min until 125 RU capture. For interaction measurements, LOS R1 or LOS R3 solubilized in DDM was injected at increasing concentrations in running buffer at 20 µL/min. Streptavidin flow cell surface was used as reference for correction of the binding response. Regeneration of the surfaces was achieved by 50 mM EDTA, pH 8. Binding curves were analyzed using Biacore T200 Evaluation Software 3.2.1 (GE Healthcare), and data were fit using steady-state affinity model.

### NMR titrations

Human ^15^N-labeled MGL-CRD^wt^ or MGL-CRD^D269H^ at 50 µM in 25 mM Tris, pH 8, 150 mM NaCl, and 4 mm CaCl_2_ was titrated with increasing concentrations of GalNAc, R1, or R3 OSs up to 20 molar equivalents of glycan:CRD. ^1^H-^15^N-BEST-TROSY correlation experiments were recorded at 30°C on an 850, 700, or 600 MHz Bruker NMR spectrometer equipped with a cryoprobe at each OS addition. NMR titration experiments with MGL-ECD were performed at a concentration of 600 µM of the ^2^H,^15^N MGL-ECD with 1 and 2 molecular equivalents of either OS R1 or R3 ligands. ^1^H-^15^N-BEST-TROSY correlation spectra were collected at 35°C on Bruker Avance spectrometer at 850 MHz. All spectra were processed using TopSpin 3.5 software and analyzed using CcpNmr analysis 3.0 software. CSPs, corresponding to the chemical shift change in the ^1^H-^15^N BTROSY spectra upon addition of ligands, were calculated as CSP = ((Δδ^1^H)^2^ + ([Δδ^15^N/10])^2^)^1/2^, where Δδ^1^H and Δδ^15^N are chemical shift changes in amide proton and amide nitrogen, respectively. CSPs higher than twice the standard deviation of all chemical shifts were considered significant.

### SAXS

SAXS data have been recorded on MGL-ECD domain at 1 mg/mL in 25 mM Tris, pH 8, 150 mM NaCl, and 4 mm CaCl_2_ buffer at 25°C at the European Synchrotron Radiation Facility (ESRF) BM29 Biosaxs beamline (Grenoble). Automatic frame selection and buffer subtraction were performed by ISPyB (38). SAXS data were analyzed with Atsas 3.1.3 (39) and BIoXTAS RAW (40). *P*(*r*) distribution function was used as input for DAMMIF online, doing five runs including P3 symmetry and prolate anisotropy. The five solutions were sorted by DAMAVER as two clusters, and the most representative envelope of the best cluster is presented. The AlphaFold multimer program was run with the entire sequence of the MGL-ECD construct expressed, and as a trimeric protein as input. Twenty-four models have been generated, and the 10 best ranked models according to their DockQ score were retained for further analysis (41).

## Supplementary Material

pgad310_Supplementary_Data

## Data Availability

All data required for main findings of this manuscript are included in the article and Supplementary material.

## Preprint

This manuscript was posted on a preprint: https://doi.org/10.1101/2023.03.02.530591.
